# Evaluating the impact of atezolizumab on febrile neutropenia occurrence in patients with NSCLC undergoing chemotherapy in Japan: a real-world post-marketing database study

**DOI:** 10.1007/s10147-024-02669-y

**Published:** 2024-12-16

**Authors:** Sayuri Nakane, Akinori Yuri, Yuki Miyano, Kana Yamada, Erika Nakatsuji, Nobuki Takei, Yasuhiro Igarashi, Ryousuke Harada

**Affiliations:** 1 Safety Science 2 Department, Drug Safety Division, Chugai Pharmaceutical Co., Ltd., 1-1 Nihonbashi-Muromachi 2-Chome, Chuo-Ku, Tokyo 103-8324 Japan; 2 Safety Science 1 Department, Drug Safety Division, Chugai Pharmaceutical Co., Ltd., Chuo-Ku, Japan; 3 Data Management Department, Drug Safety Division, Chugai Pharmaceutical Co., Ltd., Chuo-Ku, Japan

**Keywords:** Lung cancer, Non-small cell lung cancer, Atezolizumab, Febrile neutropenia, Post-marketing database study

## Abstract

**Background:**

Febrile neutropenia (FN) is a recognised adverse event associated with chemotherapy. This study investigates the impact of atezolizumab, an immune checkpoint inhibitor, on the incidence of FN in patients with non-small cell lung cancer receiving concurrent chemotherapy in Japan.

**Methods:**

This post-marketing database study was conducted using data from patients with non-small cell lung cancer provided by Medical Data Vision Co., Ltd. covering April 2008 to present. The primary outcome measured was FN incidence, and its causal association with atezolizumab use was examined by comparing the atezolizumab plus bevacizumab plus carboplatin plus paclitaxel [ABCP])-containing regimen to the BCP control group. The data period was from 1 September, 2015, to 31 December, 2021, including approval date of this drug, 21 December, 2018.

**Results:**

The database identified 301 subjects for the ABCP regimen (exposure) group, 44 for the BCP regimen (cohort design control) group during the same period, and 207 for BCP regimen (historical cohort design control) group before the approval of atezolizumab. For historical cohort design, the incidence and adjusted incidence ratios of febrile neutropenia in the exposure group to the control group were 6.13 (95% CI 2.78–13.49) and 8.19 (95% CI 3.79–25.33), respectively. Sensitivity analysis showed FN occurred in 17% (52/301) of the exposure group, 4.5% (2/44) of the cohort design control group, and 3% (7/207) of the historical cohort design control group.

**Conclusions:**

The incidence of FN was higher in the exposure group. Considering the study results, special caution is needed for FN occurrence in patients receiving atezolizumab.

**Supplementary Information:**

The online version contains supplementary material available at 10.1007/s10147-024-02669-y.

## Introduction

Lung cancer is the leading cause of cancer-related fatalities worldwide [1, 2]. Non-small cell lung cancer (NSCLC) constitutes 85–90% of the lung cancer diagnoses, with the majority of patients in advanced stages generally having less favourable prognoses [3, 4]. When considering all stages of NSCLC combined, the overall 5-year relative survival rate for NSCLC during this period (2012–2018) was 28%, underscoring the importance of early detection and treatment in improving outcomes for this type of lung cancer [5].

Febrile neutropenia (FN) is a critical medical concern, potentially emerging as a side effect of various anticancer treatments, including regimens such as bevacizumab plus carboplatin plus paclitaxel (BCP) [6, 7]. Patients with FN are at a higher risk of developing infections, necessitating prompt intervention with antibiotics and comprehensive supportive care [6]. The consequences of FN can include dose reductions, treatment delays, and a substantial impact on morbidity and mortality. Studies conducted across diverse inpatient- and outpatient-care settings have shown a 16.8% risk of developing FN during the course of chemotherapy [6, 8]. Furthermore, research indicates a possible link between immune checkpoint inhibitor (ICI) therapy and FN. Notably, FN emerged as the leading cause of death in patients receiving combined ICI and chemotherapy treatment [9].

Atezolizumab (Tecentriq^®^) is an antibody for the programmed cell death-1 ligand (PD-L1) and it exerts its efficacy by inhibiting the binding of PD-L1 to both the programmed cell death-1 (PD-1) and B7-1 receptors, thereby restoring tumour-specific immunity [10]. Therefore, it is reasonable that atezolizumab has an impact on PD-L1-expressed cancer cells [11]. The efficacy of the drug was proven in several types of cancer and, consequently, atezolizumab has indications for unresectable advanced or recurrent NSCLC, adjuvant therapy for PD-L1-positive NSCLC, advanced small cell lung cancer (SCLC), unresectable hepatocellular carcinoma (HCC), and PD-L1-positive unresectable or recurrent triple-negative breast cancer. In Japan, atezolizumab was approved for unresectable advanced or recurrent NSCLC treatment on 21 December 2018, which was the date of approval for the IMpower150 trial.

IMpower150, a pivotal phase 3, multicentre, open-label, randomised clinical trial sponsored by F. Hoffmann-La Roche/Genentech, studied the efficacy and safety of chemotherapy combinations involving atezolizumab [12]. In this study, FN was a common grade 3 or 4 treatment-related adverse event. The incidence was higher by less than 10 percentage points in the ABCP group compared to the BCP group [12]. A subgroup analysis of Japanese patients showed a higher incidence of FN (19.4% [7/36]) in the chemotherapy group involving atezolizumab (atezolizumab plus carboplatin plus paclitaxel [ACP] or atezolizumab plus bevacizumab plus carboplatin plus paclitaxel [ABCP]) compared to the control group (BCP) (4.2% [1/24]). This suggests that ABCP therapy may pose a higher risk of FN than the BCP control group. Furthermore, a case report details a patient developing transient severe neutropenia following treatment with atezolizumab. This case involving a non-Japanese patient suggests neutropenia may be a potential adverse event associated with atezolizumab therapy irrespective of race [13]. Further investigations are warranted to confirm this observation and elucidate the underlying mechanisms.

Although there are case reports in Japan about the occurrence of FN when atezolizumab is used in combination with other anticancer drugs, there have been no epidemiological database studies [14]. Based on this observation, Chugai Pharmaceutical Co., Ltd., a marketing authorisation holder in Japan, specified FN as an important potential risk in its Japanese Risk Management Plan and decided to conduct this post-marketing database study to examine the causal association between atezolizumab use and FN occurrence among patients with NSCLC. Therefore, in this study, FN incidence for ABCP treatment compared with BCP treatment in NSCLC was examined in a real-world setting using a hospital-based claims database provided by Medical Data Vision (MDV) Co., Ltd.

## Materials and methods

### Data source

A post-marketing database study was conducted using data from patients with NSCLC from a large hospital-based claims database provided by MDV Co., Ltd. (MDV; hereinafter referred to as “Database [DB]”), Tokyo, Japan. The database covers 485 acute care hospitals (about 27% of all acute care hospitals nationwide) in Japan and contains medical data from April 2008 to present, including both inpatient and outpatient records. As of August 2023, the database contained data on 44 million patients [15].

### Study design and study population

The study was implemented using both historical cohort and cohort designs. Patients who were potentially eligible for NSCLC treatment were extracted from the database and divided into an exposure group given the ABCP regimen and a control group given the BCP regimen. Furthermore, for the historical cohort design, the enrolment period for the control group was set before the approval of atezolizumab, and for the cohort design, the enrolment period for the control group was set to be the same as that for the exposure group and from the date of approval of atezolizumab. The historical cohort design was established since the treatment could be shifted from the BCP regimen to the ABCP regimen after atezolizumab approval and not enough cases in the control group could be obtained for comparison. For the exposure group of both historical cohort and cohort designs, subjects who started the ABCP regimen from 21 December 2018 (the date atezolizumab was approved for NSCLC treatment in Japan) to 20 May 2021 were selected. In the cohort design control group, subjects who started the BCP regimen within the same period in which the exposure group started were selected; on the other hand, for the control group in the historical cohort design, subjects who started the BCP regimen from 21 December 2015 to 20 May 2018 were selected.

**Fig. 1:**
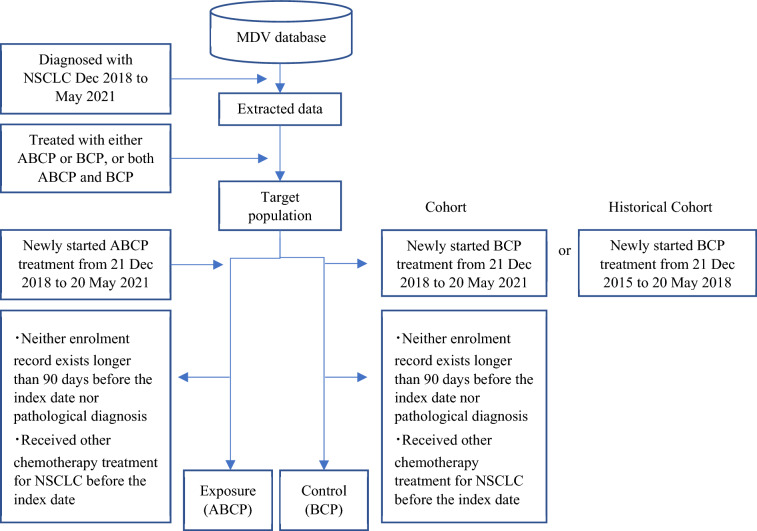
Overview of patient selection process for historical cohort and cohort design groups. *ABCP* atezolizumab plus bevacizumab plus carboplatin plus paclitaxel, *BCP* bevacizumab plus carboplatin plus paclitaxel, *MDV* medical data vision, *NSCLC* non-small cell lung cancer

International Classification of Diseases 10th revision (ICD-10) codes and receipt codes used to identify subjects were presented in Supplementary Table 1 and Supplementary Table 2, respectively. An overview of the patient selection process is presented in Fig. 1.

### Outcomes

The outcome definitions were based on the treatments given to patients determined to be at high risk during initial testing in the FN clinical practice guidelines and defined based on the combination of: (i) disease name—must have FN (disease name code: 8842350) and (ii) treatment—must follow treatment (intravenous antimicrobial agent) when FN develops. The onset date was defined as the first day of the antibiotic prescription. Prespecified antimicrobial beta-lactam agents (Anatomical therapeutic chemical [ATC] codes for antimicrobial) were defined, as they were experimentally prescribed for FN treatment in Japan (Supplementary Table 3).

### Observation period

The observation period started from the index date, which was defined as the date of the first administration of the regimen. The observation period ended at the earliest of: (I) 180 days after the index date; (II) the preceding day of the regimen switch; (III) the end of treatment; (IV) the last day of the dataset; and (V) the date of outcome onset. Each observation period at risk for the subject was summed to yield the incidence rate.

### Covariates

The covariates included in the analysis were identified based on their potential influence on both treatment selection and the occurrence of the outcome, as outlined in the FN clinical practice guideline [16, 17]. These covariates were selected from variables available within the database (Supplementary Table 4). Where the propensity scores could not be calculated appropriately due to the number of cases in the exposure or control group or the bias of individual covariates in both groups, considering the data distribution and clinical importance, one of the covariates was calculated. In the case of covariates that were excluded due to the aforementioned reasons, the impact of those exclusions was taken into consideration when interpreting the results.

### Sensitivity analyses

For the sensitivity analysis a method used to evaluate how different assumptions or analyses can impact the predetermined research questions [18], analyses were performed (1) with a changed definition of the observation period end date, (2) with a changed definition of outcomes, (3) excluding cases with less than 90 days of data enrolment before the observation start date and chemotherapy treatment for NSCLC. To evaluate the robustness of the analysis, a sensitivity analysis was conducted to account for potentially excluded cases due to these criteria, including patients transferred to other hospitals, (4) with trend score trimming.

### Statistical analysis

SAS Version 9.4 (SAS Institute, Inc., Cary, NC, USA) was used for this study.

### Propensity scores

The propensity score for each subject was calculated by a logistic model in which the dependent variable was the group of drugs administered (exposure or control group) and the independent variables were the covariates of the subject background. The obtained propensity score distribution was confirmed by a histogram.

### Standardised differences

The balance of covariates between the exposure and control groups was confirmed by a standardised difference calculated for each patient covariate using the following formula:

$$\text{Standardised difference}({\%})=100\times \frac{\text{difference of mean between exposure and control groups }}{\text{standard deviation of entire population}}.$$

### Standardised morbidity ratio weights (SMRW)

Each variable in the control group was weighted by exposure odds calculated by the propensity score, which was applied to produce a virtual control group for adjusting to the exposure group.

### Incidence rates and 95% confidence intervals

The incidence rates per person-year and their 95% confidence intervals were calculated using the following formula:

The total observation period was estimated by the Kaplan–Meier method:

$$\text{incidence rate per person-year}=\frac{\text{number of first outcomes in the group}}{\text{total observation period}/365}$$

$$95\%\text{ confidential interval}=\text{ incidence rate per person-year}\pm 1.96\sqrt{\frac{\text{number of first outcomes in the group}}{(\text{total observation period}/365)^{2}}}.$$

## Results

### Study population

**Fig. 2:**
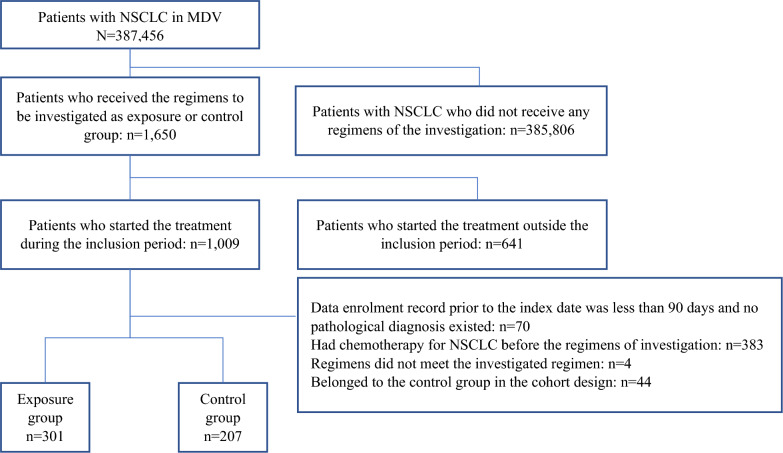
Patient configuration for historical cohort design group. *MDV* medical data vision, *NSCLC* non-small cell lung cancer

From the MDV data, 301 subjects in the exposure group and 44 subjects in the control group were selected to comprise the cohort design (Supplementary Fig. 1), and 207 subjects in the control group were selected to comprise the historical cohort design (Fig. 2). Since the number of cases in the control group in the historical cohort design was greater than that for the cohort design, the analysis was based on the results of the historical cohort design.

### Patient demographics before and after weighting by SMRW

The demographics and clinical characteristics of the subjects obtained before and after weighting by SMRW are shown in Table 1 for the historical cohort design, and in Supplementary Table 5 for the cohort design.

**Table 1:** Patient background (historical cohort design)

		Before correction		Normalisation difference	After correction		Normalisation difference
		Exposure group	Control group		Exposure group	Control group	
Total n (%)		301 (100.0%)	207 (100.0%)	–	301.0 (100.0%)	306.2 (100.0%)	
Sex	Male	219 (72.8%)	151 (72.9%)	− 0.004	219.0 (72.8%)	240.3 (78.5%)	− 0.133
Age	Mean ± SD	65.0 ± 9.4	66.6 ± 8.5	− 0.176	65.0 ± 9.4	65.5 ± 10.4	− 0.052
	15 years	0	0	–	0	0	–
	15 years ≤ , < 65 years	117 (38.9%)	70 (33.8%)	0.105	117.0 (38.9%)	132.0 (43.1%)	− 0.086
	65 years ≤	184 (61.1%)	137 (66.2%)	− 0.105	184.0 (61.1%)	174.2 (56.9%)	0.086
Barthel Index	N	289	195	–	289.0	286.1	–
	Mean ± SD	96.7 ± 12.3	97.4 ± 11.0	− 0.066	96.7 ± 12.3	96.8 ± 12.7	− 0.014
Clinical classification	Hospitalisation	90 (29.9%)	38 (18.4%)	0.272	90.0 (29.9%)	77.3 (25.2%)	0.105
	Outpatient	2 (0.7%)	9 (4.3%)	− 0.237	2.0 (0.7%)	6.0 (2.0%)	− 0.115
	Inpatient and outpatient	209 (69.4%)	160 (77.3%)	− 0.179	209.0 (69.4%)	222.9 (72.8%)	− 0.074
Clinical classification (at start of dose)	Hospitalisation	298 (99.0%)	195 (94.2%)	0.267	298.0 (99.0%)	298.5 (97.5%)	0.117
Medical history	Yes	9 (3.0%)	7 (3.4%)	− 0.022	9.0 (3.0%)	8.7 (2.9%)	0.008
Renal dysfunction	Yes	3 (1.0%)	6 (2.9%)	− 0.138	3.0 (1.0%)	1.7 (0.5%)	0.051
Liver dysfunction	Yes	28 (9.3%)	15 (7.2%)	0.075	28.0 (9.3%)	33.5 (10.9%)	− 0.054
Infectious diseases	Yes	9 (3.0%)	7 (3.4%)	− 0.022	9.0 (3.0%)	8.7 (2.9%)	0.008
Neutropenia	Yes	0	0	–	0	0	–
FN	Yes	0	0	–	0	0	–
History of surgery for the underlying disease	Yes	4 (1.3%)	14 (6.8%)	− 0.278	4.0 (1.3%)	3.9 (1.3%)	0.005
History of surgery for non-primary disease	Yes	44 (14.6%)	36 (17.4%)	0.076	44.0 (14.6%)	43.2 (14.1%)	0.015
Pre-therapeutic radiotherapy	Yes	28 (9.3%)	16 (7.7%)	0.056	28.0 (9.3%)	26.3 (8.6%)	0.025
Combined radiotherapy	Yes	16 (5.3%)	9 (4.3%)	0.045	16.0 (5.3%)	10.7 (3.5%)	0.088
Pre-treatment (G-CSF preparation)	Yes	0 (0.0%)	2 (1.0%)	− 0.140	0.0 (0.0%)	0.0 (0.0%)	0.000
Pre-treatment (antimicrobial)	Yes	10 (3.3%)	6 (2.9%)	0.024	10.0 (3.3%)	7.0 (2.3%)	0.062
Previous treatment (anticancer drugs other than lung cancer)	Yes	1 (0.3%)	6 (2.9%)	− 0.205	1.0 (0.3%)	0.8 (0.3%)	0.011
Prior treatment (EGFR inhibitor)	Yes	62 (20.6%)	18 (8.7%)	0.342	62.0 (20.6%)	75.6 (24.7%)	− 0.098
Prior treatment (ALK inhibitor)	Yes	5 (1.7%)	1 (0.5%)	0.115	5.0 (1.7%)	0.0 (0.0%)	0.184
Pre-treatment (immune checkpoint inhibitor)	Yes	0 (0.0%)	4 (1.9%)	− 0.199	0.0 (0.0%)	0.0 (0.0%)	0.000
Total duration (days)	Mean ± SD	72.8 ± 41.7	87.3 ± 49.8	− 0.316	72.8 ± 41.7	84.0 ± 61.9	− 0.213
Total number of cycles	Mean ± SD	2.8 ± 1.4	3.1 ± 1.6	− 0.192	2.8 ± 1.4	3.0 ± 2.1	− 0.113
Number of cycles	1	94 (31.2%)	57 (27.5%)	0.081	94.0 (31.2%)	92.8 (30.3%)	0.02
	2	32 (10.6%)	21 (10.1%)	0.016	32.0 (10.6%)	37.8 (12.3%)	− 0.053
	3	35 (11.6%)	29 (14.0%)	− 0.071	35.0 (11.6%)	43.0 (14.0%)	− 0.072
	4	128 (42.5%)	70 (33.8%)	0.18	128.0 (42.5%)	84.5 (27.6%)	0.317
	5	5 (1.7%)	6 (2.9%)	− 0.083	5.0 (1.7%)	6.4 (2.1%)	− 0.031
	6 or more	7 (2.3%)	24 (11.6%)	− 0.37	7.0 (2.3%)	41.9 (13.7%)	− 0.428
Bevacizumab total dose	Mean ± SD	2541.2 ± 1435.2	2775.6 ± 1610.6	− 0.154	2541.2 ± 1435.2	2748.1 ± 2093.7	− 0.115
Total carboplatin dose	Mean ± SD	1575.4 ± 937.4	1687.2 ± 1076.2	− 0.111	1575.4 ± 937.4	1732.4 ± 1458.7	− 0.128
Paclitaxel total dose	Mean ± SD	821.1 ± 449.9	831.2 ± 541.2	− 0.020	821.1 ± 449.9	859.7 ± 743.9	− 0.063
Combination steroids	Yes	301 (100.0%)	207 (100.0%)	–	301.0 (100.0%)	306.2 (100.0%)	–
Combined steroid dose (mg)	N	301	207	–	301.0	306.2	–
	Mean ± SD	716.9 ± 864.2	762.4 ± 1301.3	− 0.041	716.9 ± 864.2	653.5 ± 1322.3	0.057

*ALK *anaplastic lymphoma kinase, *EGFR* epithelial growth factor receptor, *FN* febrile neutropenia, *G-CSF* granulocyte colony stimulator, *N* number of cases, *SD* standard deviation

**Fig. 3:**
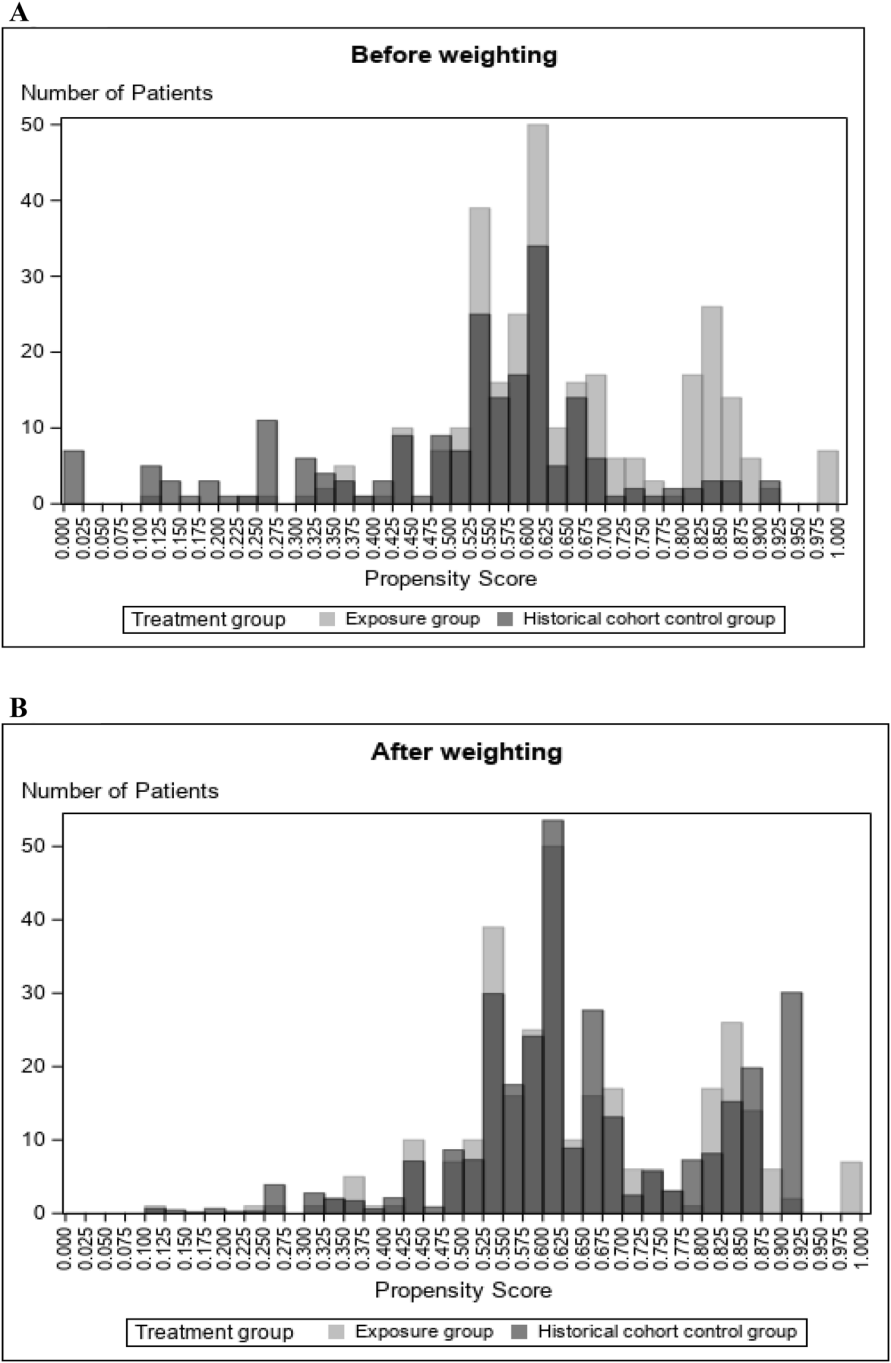
Distribution of propensity score in exposure and control groups for the historical cohort design group A before weighting and B after weighting

To investigate the atezolizumab effect on FN in patients with NSCLC, we weighted the patient values in the control group by the exposure odds calculated by the propensity score (SMRW) to balance the patient background. When calculating the propensity scores using all the listed covariates in the Materials and Methods section, the estimation of the propensity score did not converge. This is because there are combinations of highly correlated items that, in some cases, are biased towards one category (listed in Supplementary Table 6). Therefore, the propensity score was calculated using the covariates by considering clinical significance. The distribution of the propensity scores in the exposure and control groups is presented in Fig. 3A before weighting and Fig. 3B after weighting for the historical cohort design, and in Supplementary Fig. 2A before weighting and Supplementary Fig. 2B after weighting for the cohort design.

The subject characteristics in historical cohort design group before weighting by SMRW were similar in both the exposure group and the control group; however, the length of treatment was slightly longer in the control group (87.3 ± 49.8 days in the control group and 72.8 ± 41.7 days in the exposure group). The standardised difference improved to less than 0.2 after weighting in all the covariates, except for the length of treatment.

### FN occurrence and incidence rate in exposure and control groups

The occurrence and incidence rate per person-year of FN among patients with NSCLC for the cohort design (Supplementary Table 6) and historical cohort design **(**Table 2) were examined. For the historical cohort design, the FN incidence rate was 0.87 (95% confidence interval [CI] 0.63–1.10) per person-year in the exposure group and 0.14 (95% CI 0.04–0.25) in the control group. The number of cases in the exposure group, the cohort design control group, and the historical cohort design control group was 301, 44, and 207, respectively. FN developed during the observation period among 52, 2, and 7 patients in the exposure group, cohort design control group, and historical cohort design control group, respectively. For the cohort design, the incidence ratios of FN in the exposure group to the control group and the corrected incidence ratio for the pseudo-population generated by the SMRW method were 4.70 (95% CI 1.15–19.30) and 7.89 (95% CI 2.59–2.12 × 10^11^), respectively. The upper limit of the confidence interval from the bootstrap method was output as large because the number of outcomes in the control group was 0. For the historical cohort design, the incidence and adjusted incidence ratios of FN in the exposure group to the control group were 6.13 (95% CI 2.78–13.49) and 8.19 (95% CI 3.79–25.33), respectively. A higher incidence was observed in the exposure group, even after adjustment, using the pseudo-cohort generated by SMRW.

**Table 2:** Differences in incidence and adjusted incidence of febrile neutropenia in historical cohort design group

	Exposure group	Control group
Number of subjects	301	207
Number of FN occurrences	52	7
Incidence rate		
Incidence rate per person-year	0.87 (0.63–1.10)	0.14 (0.04–0.25)
Incidence rate ratio (crude)	6.13 (2.78–13.49)	
Incidence rate ratio (adjusted)	8.19 (3.79–25.33)	
Incidence rate difference (crude)	0.73 (0.47–0.98)	
Incidence rate difference (adjusted)	0.76 (0.51–1.03)	
Occurrence rate		
Occurrence rate (× 100%)	0.17 (0.13–0.22)	0.03 (0.01–0.06)
Occurrence rate ratio (crude)	5.11 (2.37–11.02)	
Occurrence rate ratio (adjusted)	7.10 (3.24–20.90)	

*FN* febrile neutropenia

FN occurrence was further examined by sensitivity analysis. The exposure group have a higher risk of FN among the NSCLC population.

### Difference in FN incidence regarding NSCLC treatment

As a higher FN risk rate in the atezolizumab-containing regimen compared to the atezolizumab non-containing regimen was observed, the difference in the FN incidence rate was investigated to gain further insight into the impact of atezolizumab on NSCLC treatment. The incidence rate difference was 0.73 (95% CI: 0.47–0.98) per person-year before the adjustment and 0.76 (95% CI: 0.51–1.03) after the adjustment **(**Table 2).

### The period until the occurrence of FN after correction in each drug group

**Fig. 4:**
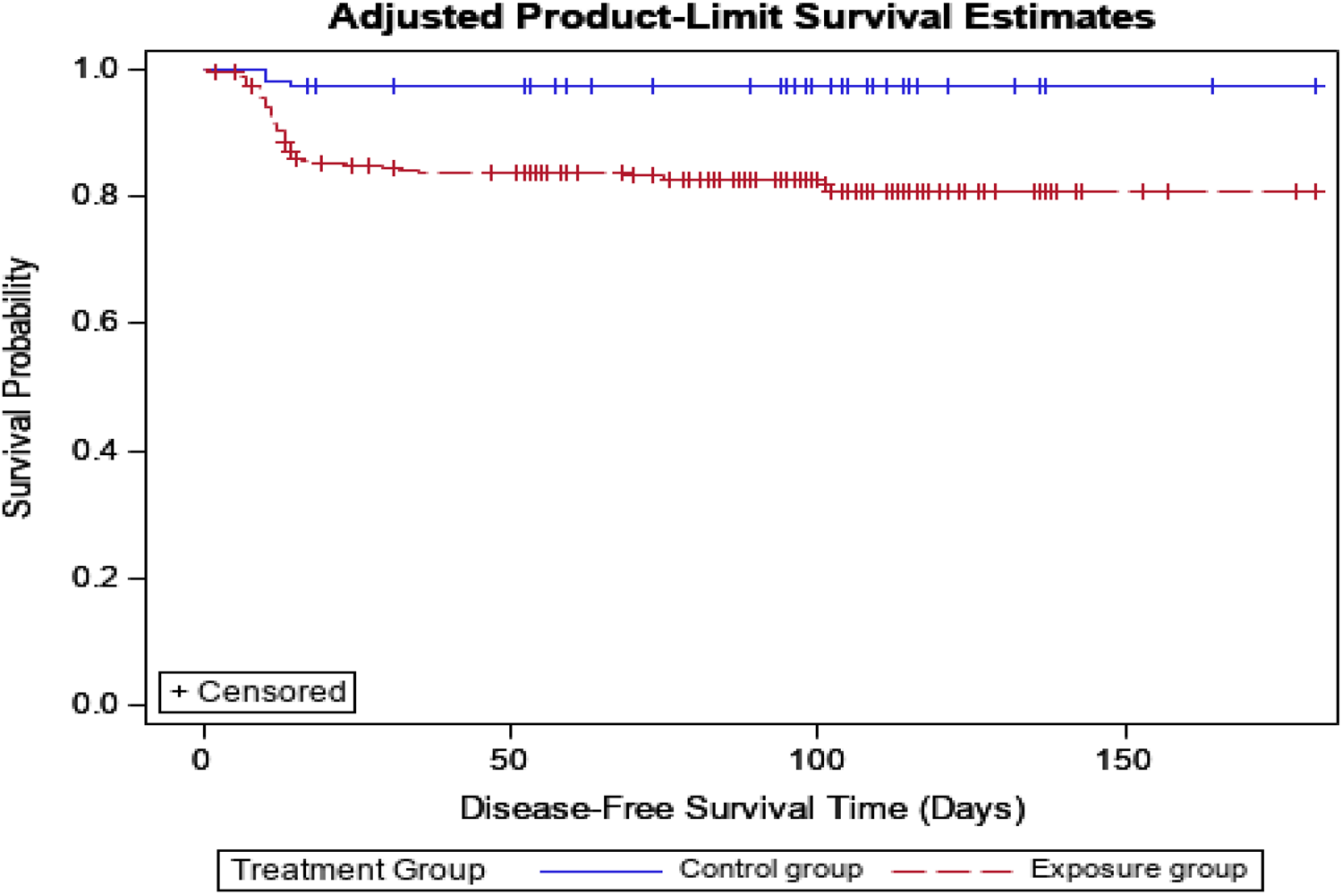
Kaplan–Meier curves for time to FN onset (cohort design). *FN* Febrile neutropenia

The Kaplan–Meier (KM) curve in Fig. 4 shows the adjusted time until FN occurs in both the exposure and control groups of the cohort study. For each time point (starting from the date of administration and every 30 days thereafter), the number of individuals at risk, cumulative cases of FN, and non-FN incidence rates are detailed. Quartile estimates (with 95% CI) for the time until FN occurrence is included, but since less than 75% of participants experienced FN, quartile data for FN occurrence times was not calculated. The KM analysis shows that the exposure group experienced a steady decline in the rate of non-occurrence of outcomes, dropping from 84.54% (95% CI 80.43–88.39) at Day 30 to 80.87% (95% CI 76.13–86.20) by Day 180. In contrast, the control group maintained a high non-occurrence rate of 97.31% from Day 30 (95% CI 92.42–100.00) to Day 180 (95% CI 92.59–100.00), indicating a higher rate of non-occurrence of outcomes compared to the exposure group throughout the study period.

## Discussion

Our study findings suggest that there is a risk of developing FN in the cohort receiving atezolizumab. In the IMagyn050 trial, the only significant adverse event, regardless of investigator-assessed causality, occurring in 2% or more of patients in either group was FN (8% for atezolizumab and 4% for placebo) [19]. Atezolizumab, a monoclonal antibody, exerts its mechanism by binding to PD-L1, disrupting PD-L1/PD-1 interaction and consequently augmenting T-cell activity directed against neoplastic cells [20]. In a meta-analysis, Petrelli et al. examined patients treated with PD-L1 inhibitors across various tumour types. Among 9324 patients from 47 studies, they reported an FN incidence of 0.45% [21]. To address FN, primary prophylaxis with granulocyte colony stimulating factor (G-CSF) preparations based on the frequency of FN onset and empiric treatment of FN are necessary, and it is important to carry out evaluations by repeated monitoring of blood cultures and neutrophils 3–4 days after starting the empiric treatment [16, 17]. In addition, to reduce the progression of FN, it is recommended to take preventive measures such as conducting regular blood tests and carefully monitoring the patient’s condition while administering atezolizumab [22].

Recent studies have investigated the safety of atezolizumab-containing regimens, highlighting the incidence of FN as an adverse effect. For example, a study conducted by Hata et al. compared the safety of ABCP with that of the BCP combination for treating advanced non-squamous (NSQ)-NSCLC in Japanese patients, observing higher incidences of FN in the ABCP group [23]. Similarly, Shiraishi et al. compared the safety of atezolizumab and platinum plus pemetrexed, with or without bevacizumab, for metastatic NSQ-NSCLC in Japanese patients, noting higher incidences of FN in the atezolizumab-containing regimens, with FN rates of 10% vs 6% (*P* = 0.15) among patients treated with atezolizumab, carboplatin plus pemetrexed, and bevacizumab vs atezolizumab plus carboplatin with pemetrexed [24]. In addition, Amari et al. evaluated the safety and patient-reported outcomes of atezolizumab plus chemotherapy, with or without bevacizumab, in stage IIIB/IV NSQ-NSCLC patients, finding higher incidences of adverse events, particularly neutropenia (19.1% in platinum-pemetrexed-atezolizumab-bevacizumab; 23.6% in platinum-pemetrexed-atezolizumab.) [25]. Endo et al. analysed the adverse event profile of the ABCP combination therapy based on Japanese Adverse Drug Event Report data, emphasising the importance of effectively managing FN and skin-related adverse events [26]. Although the indications and regimen are not the same as in this study, the incidence of FN tends to be high, increasing the robustness of the results. All these studies highlight the increased risk of adverse events, including FN, with atezolizumab-containing regimens.

The growing use of ICIs in diverse cancer treatments has heightened the detection of rare immune-related adverse events (irAE). Among these, immune-related neutropenia, although infrequent, poses a significant risk of mortality due to its potential to lead to sepsis. Recognising and promptly addressing haematological irAE are vital for ensuring favourable outcomes in patients undergoing ICI therapy [27]. The mechanism of action for the increase in FN when atezolizumab is combined with other factors is not fully understood. FN can occur as a side effect for a few cancer treatments, including immunotherapies such as atezolizumab. It is believed to result from the suppression of the immune system, particularly the neutrophil count, which can leave the patient vulnerable to infections. Atezolizumab use, when combined with other patient-related risk factors, such as age, comorbidities, and prior chemotherapy, may further affect the immune system, potentially increasing the risk of FN [6, 7]. However, the specific mechanisms and interactions leading to this increased risk may vary depending on the context and individual patient characteristics, and further research is needed to fully elucidate this process. In addition, the genetic factors contributing to FN can vary among individuals, and there may be a few differences in FN susceptibility between populations in Japan and elsewhere. While there may not be a single conclusive genetic factor, several genetic variations can influence the risk of FN in patients undergoing chemotherapy, such as variations in genes responsible for drug metabolism, immune response, and the production of blood cells [28]. Comparing the frequency of FN between patients in Japan and other regions could potentially reveal differences in the prevalence of these genetic factors. To understand these genetic factors and their interactions, studies involving large cohorts from both regions would be necessary. Researchers would need to investigate specific genetic markers and variations that may predispose certain individuals to FN when undergoing chemotherapy. This research could help identify genetic factors that contribute to the varying frequencies of FN between different populations and inform personalised treatment approaches to reduce the FN risk.

The limitations of this study stem from the medical information database utilised. This database contains data from visits to specific medical institutions. Consequently, patients receiving anticancer drugs for NSCLC at the same institution were excluded based on predefined criteria. However, if a patient had prior anticancer treatment elsewhere, the data might only reflect cases occurring after second-line treatment. In addition, relevant events or outcomes from other medical facilities cannot be accurately identified, including the patient’s medical history, surgical history, radiotherapy, and prior treatment. Outcome validation was not performed, limiting the interpretation of point estimates such as incidence rates. Propensity scores could not be adjusted due to limited control cases and uneven group distribution. In addition, the database, collected for insurance claims, lacks information on non-essential items. This analysis only considers beta-lactams based on treatment guidelines [29]. Other antibiotics, like new quinolones, may be used in practice. Furthermore, in this study, the total dose of Carboplatin and Paclitaxel was recorded for each patient group, but the total dose per dose was not calculated. Therefore, it cannot be denied that the high dose per dose may have contributed to the high incidence of FN. However, Table 1 shows the mean and standard deviation (SD) of the number of cycles and drug administration. While there was no general difference in the distribution of the number of cycles between the exposure group and the control group, the total dose in the exposure group was small and the incidence of FN was higher than in the control group, so it is likely that the dose per dose did not contribute. The current study design cannot fully distinguish the independent contribution of neutropenia to the observed outcomes from the potential confounding effect of fever. The high co-occurrence of fever and FN makes it challenging to isolate the specific effects of neutropenia on the investigated parameters. Future studies employing stratification or matching based on the presence of fever may be necessary to definitively attribute the observed effects to neutropenia alone. Lastly, relying on the receipt database for FN identification may underestimate prevalence. The absence of neutrophil count data and the potential for missed cases due to terminology limitations are key constraints. In addition, since the database used in this survey does not allow for the acquisition of clinical test values, it was not possible to define outcomes using clinical test values.

As mentioned before, an elevated FN occurrence was observed with atezolizumab plus bevacizumab plus chemotherapy in patients with metastatic NSCLC in the IMpower150 clinical trial [12]. Although this international phase III clinical trial was conducted among a limited number of Japanese patients in a global population and direct comparisons were not possible due to differences in patient backgrounds with this DB study, there were a few similarities between these 2 studies. The IMpower150 trial showed a higher incidence (19.4%) of FN with atezolizumab involving chemotherapy in the ACP or ABCP group than in the control BCP group (4.2%). This clearly demonstrates that FN is a concern in both studies, with higher incidence rates noted in exposed groups.

To the best of our knowledge, the present DB study represents a pioneering effort in exploring the association between atezolizumab and the incidence of FN in a real-world context, using a comprehensive Japanese medical records database. While acknowledging the existence of the IMpower150 trial, which shares certain similarities with our DB study, it is important to note that, apart from this trial, no other investigations of comparable scope and nature have been conducted. Thus, this study contributes valuable insights into the real-world implications of atezolizumab use in relation to FN occurrence, filling a notable gap in the existing research landscape.

## Supplementary Information

Below is the link to the electronic supplementary material.

Supplementary file1 (DOCX 304 KB)

## Data Availability

The authors confirm that all data analysed during this study are included in this manuscript and supplementary document.
