# Supplementary material for: Evaluating the impact of atezolizumab on febrile neutropenia occurrence in patients with NSCLC undergoing chemotherapy in Japan: a real-world post-marketing database study
Source: Int J Clin Oncol. 2024 Dec 16;30(2):298–308. doi: 10.1007/s10147-024-02669-y (PMC11785710; doi:10.1007/s10147-024-02669-y)

## **Supplementary Document**

**Title:** Evaluating the Impact of Atezolizumab on Febrile Neutropenia Occurrence in Patients with NSCLC Undergoing Chemotherapy in Japan: A Real-World Post-Marketing Database Study

**Author List**

Sayuri Nakane**^1^**, Akinori Yuri**^1^**, Yuki Miyano**^1^**, Kana Yamada**^1^**, Erika Nakatsuji**^1^**, Nobuki Takei**^1^**, Yasuhiro Igarashi**^2^**, Ryousuke Harada**^3^**

Author Affiliations:

*^1^ Safety Science 2 Department, Drug Safety Division, Chugai Pharmaceutical Co., Ltd.*

*^2^ Safety Science 1 Department, Drug Safety Division, Chugai Pharmaceutical Co., Ltd.*

*^3^ Data Management Department, Drug Safety Division, Chugai Pharmaceutical Co., Ltd.*

**Corresponding Author**

Sayuri Nakane ([nakane.sayuri35@chugai-pharm.co.jp](mailto:nakane.sayuri35@chugai-pharm.co.jp))

Chugai Pharmaceutical Co., Ltd.

1-1 Nihonbashi-Muromachi 2-Chome, Chuo-Ku, Tokyo, Japan, 103-8324

## **Supplementary Tables**

Supplementary Table 1. NSCLC code list

| ICD10 code | Disease code | Disease name |
| --- | --- | --- |
| C340 | 8842835 | hilar lung cancer |
| C340 | 8847676 | hilar adenocarcinoma |
| C340 | 8847677 | hilar large cell cancer |
| C340 | 8847678 | hilar non-small cell cancer |
| C340 | 8847679 | hilar squamous cell carcinoma |
| C341 | 8835493 | lung cancer of superior lobe |
| C341 | 8847634 | superior lobe lung adenocarcinoma |
| C341 | 8847635 | superior lobe large cell lung cancer |
| C341 | 8847636 | superior lobe squamous cell carcinoma of lung |
| C341 | 8847637 | superior lobe non-small cell lung cancer |
| C342 | 8837666 | lung cancer of middle lobe |
| C342 | 8847661 | middle lobe lung adenocarcinoma |
| C342 | 8847662 | middle lobe large cell lung cancer |
| C342 | 8847663 | middle lobe squamous cell lung cancer |
| C342 | 8847664 | middle lobe non-small cell lung cancer |
| C343 | 8831458 | lung cancer of inferior lobe |
| C343 | 8847595 | inferior lobe lung adenocarcinoma |
| C343 | 8847596 | inferior lobe large cell lung cancer |
| C343 | 8847597 | inferior lobe squamous cell carcinoma of lung |
| C343 | 8847598 | inferior lobe non-small cell lung cancer |
| C348 | 8833932 | bronchiolo-alveolar cell carcinoma |
| C349 | 1629003 | primary lung cancer |
| C349 | 1629006 | lung cancer |
| C349 | 1629009 | pulmonary sarcoma |
| C349 | 8838805 | pulmonary blastoma |
| C349 | 8838844 | lung adenocarcinoma |
| C349 | 8838852 | large cell lung cancer |
| C349 | 8838898 | squamous cell carcinoma of lung |
| C349 | 8838901 | alveolar cell carcinoma |
| C349 | 8842053 | non-small cell lung cancer |
| C349 | 8842831 | lung carcinosarcoma |
| C349 | 8842832 | adenosquamous carcinoma of the lung |
| C349 | 8842833 | pulmonary adenoid cystic cancer |
| C349 | 8842834 | pulmonary mucoepidermoid carcinoma |
| C349 | 8847272 | ALK fusion gene positive non-small cell lung cancer |
| C349 | 8847732 | EGFR mutation positive advanced non-small cell lung cancer |
| C349 | 8849238 | ROS1 fusion gene positive non-small cell lung cancer |
| C349 | 8849788 | adult type pulmonary blastoma |
| D022 | 2312002 | pulmonary carcinoma in situ |
| D381 | 2391024 | pulmonary tumour |

Abbreviations: ALK = anaplastic lymphoma kinase; EGFR = epithelial growth factor receptor; NSCLC = Non-small cell lung cancer; ICD10 = International Classification of Diseases 10th revision; ROS1 = ROS proto-oncogene 1.

Supplementary Table 2. Receipt code list

| Item | | Code |
| --- | --- | --- |
| Anatomical therapeutic chemical (ATC) | Bevacizumab | L01FG01 |
|  | Carboplatin | L01XA02 |
|  | Paclitaxel | L01CD01 |
|  | Atezolizumab (1,200-mg package only) | L01FF05 |

Supplementary Table 3. ATC codes of antimicrobial

| Item | Code |
| --- | --- |
| imipenem hydrate / sodium cilastatin | J01DH51 |
| cefepime hydrochloride hydrate | J01DE01 |
| cefozopran hydrochloride | J01DE03 |
| ceftazidime hydrate | J01DD02 |
| cefpirome sulphate | J01DE02 |
| sodium tazobactam / piperacillin sodium | J01CR05 |
| doripenem hydrate | J01DH04 |
| panipenem betamipron | J01DH55 |
| biapenem | J01DH05 |
| meropenem hydrate | J01DH02 |

Abbreviations: ATC = Anatomical therapeutic chemical

Supplementary Table 4. Covariates

| Category | Item | | | Code |
| --- | --- | --- | --- | --- |
| Demographic background | Sex | | | - |
|  | Age | | | - |
| Medical background | Body mass index, | | | - |
|  | Barthel Index score, | | | - |
|  | Recent medical history | renal dysfunction | | N17 to N19 |
|  |  | liver dysfunction | | K70 to K76 |
|  |  | infection | | A010 to A93, B00 to B99 |
|  |  | neutropenia | | D070 |
|  |  | FN | | - |
|  | Recent surgical history | primary disease | | K514 |
|  |  | other disease | | kubun code "K” |
|  | Recent history of radiation therapy | | | M001 to M004, |
|  | Recent medication history | antimicrobial agent | | A07, J01, J05, L03 |
|  |  | anticancer agent other than NSCLC treatment [L01 and L02], | epidermal growth factor receptor inhibitors | cetuximab (L01FE01) |
|  |  |  |  | necitumumab (L01FE03) |
|  |  |  |  | panitumumab (L01FE02) |
|  |  |  |  | afatinib (L01EB03) |
|  |  |  |  | erlotinib (L01EB02) |
|  |  |  |  | osimertinib (L01EB04) |
|  |  |  |  | gefitinib (L01EB01) |
|  |  |  |  | dacomitinib (L01EB07), |
|  |  |  |  | lapatinib (L01EH01) |
|  |  |  | anaplastic lymphoma kinase inhibitors | alectinib (L01ED03) |
|  |  |  |  | crizotinib (L01ED01) |
|  |  |  |  | ceritinib (L01ED02) |
|  |  |  |  | lorlatinib (L01ED05) |

Abbreviations: FN = Febrile neutropenia; NSCLC = Non-small cell lung cancer.

Supplementary Table 5. Patient background (cohort design)

|  | | Before correction | | Normalisation difference | After correction | | Normalisation difference |
| --- | --- | --- | --- | --- | --- | --- | --- |
|  |  | Exposure group | Control group |  | Exposure group | Control group |  |
| Total n (%) | | 301 (100.0%) | 44 (100.0%) | - | 301.0 (100.0%) | 256.1 (100.0%) | - |
| Sex | Male | 219 (72.8%) | 27 (61.4%) | 0.244 | 219.0 (72.8%) | 189.5 (74.0%) | -0.029 |
|  | Female | 82 (27.2%) | 17 (38.6%) | -0.244 | 82.0 (27.2%) | 66.5 (26.0%) | 0.029 |
| Age | N. | 301 | 44 | - | 301.0 | 256.1 | - |
|  | Mean +/- SD | 65.0 +/- 9.4 | 67.7 +/- 8.9 | -0.297 | 65.0 +/- 9.4 | 67.2 + 19.9 | -0.144 |
|  | Median (Min; Max) | 67.0 (25;82) | 69.5 (36;81) | - | 67.0 (25;82) | 68.0 (36;81) | - |
|  | 15 years | 0 | 0 | - | 0 | 0 | - |
|  | 15years ≤, <65years | 117 (38.9%) | 12 (27.3%) | 0.248 | 117.0 (38.9%) | 87.7 (34.2%) | 0.096 |
|  | 65years ≤ | 184 (61.1%) | 32 (72.7%) | -0.248 | 184.0 (61.1%) | 168.4 (65.8%) | -0.096 |
| BMI | N. | 294 | 43 | - | 294.0 | 248.8 | - |
|  | Mean +/- SD | 22.24 +/- 3.37 | 22.70 +/- 3.98 | -0.125 | 22.24 +/- 3.37 | 22.14 +/- 7.45 | 0.016 |
| Barthel Index | N. | 289 | 43 | - | 289.0 | 248.8 | - |
|  | Mean +/- SD | 96.7 +/- 12.3 | 97.9 +/- 8.7 | -0.117 | 96.7 +/- 12.3 | 99.5 +/- 10.6 | -0.245 |
| Clinical classification | Hospitalisation | 90 (29.9%) | 5 (11.4%) | 0.471 | 90.0 (29.9%) | 30.1 (11.8%) | 0.459 |
|  | Outpatient | 2 (0.7%) | 1 (2.3%) | -0.134 | 2.0 (0.7%) | 4.1 (1.6%) | -0.090 |
|  | Inpatient and outpatient | 209 (69.4%) | 38 (86.4%) | -0.417 | 209.0 (69.4%) | 221.8 (86.6%) | -0.425 |
| Clinical classification (at start of dose) | Hospitalisation | 298 (99.0%) | 42 (95.5%) | 0.217 | 298.0 (99.0%) | 247.4 (96.6%) | 0.163 |
|  | Outpatient | 3 (1.0%) | 2 (4.5%) | -0.217 | 3.0 (1.0%) | 8.7 (3.4%) | -0.163 |
| Medical history | None (90+ days) | 173 (57.5%) | 28 (63.6%) | -0.126 | 173.0 (57.5%) | 140.6 (54.9%) | 0.052 |
|  | None (less than 90 days) | 119 (39.5%) | 16 (36.4%) | 0.065 | 119.0 (39.5%) | 115.5 (45.1%) | -0.113 |
|  | Yes | 9 (3.0%) | 0 (0.0%) | 0.248 | 9.0 (3.0%) | 0.0 (0.0%) | 0.248 |
| Renal dysfunction | None (90+ days) | 176 (58.5%) | 26 (59.1%) | -0.013 | 176.0 (58.5%) | 137.9 (53.9%) | 0.093 |
|  | None (less than 90 days) | 122 (40.5%) | 16 (36.4%) | 0.086 | 122.0 (40.5%) | 115.5 (45.1%) | -0.092 |
|  | Yes | 3 (1.0%) | 2 (4.5%) | -0.217 | 3.0 (1.0%) | 2.7 (1.0%) | -0.005 |
| Liver dysfunction | None (90+ days) | 155 (51.5%) | 25 (56.8%) | -0.107 | 155.0 (51.5%) | 124.1 (48.5%) | 0.061 |
|  | None (less than 90 days) | 118 (39.2%) | 15 (34.1%) | 0.106 | 118.0 (39.2%) | 109.8 (42.9%) | -0.075 |
|  | Yes | 28 (9.3%) | 4 (9.1%) | 0.007 | 28.0 (9.3%) | 22.1 (8.6%) | 0.023 |
| Infectious diseases | None (90+ days) | 173 (57.5%) | 28 (63.6%) | -0.126 | 173.0 (57.5%) | 140.6 (54.9%) | 0.052 |
|  | None (less than 90 days) | 119 (39.5%) | 16 (36.4%) | 0.065 | 119.0 (39.5%) | 115.5 (45.1%) | -0.113 |
|  | Yes | 9 (3.0%) | 0 (0.0%) | 0.248 | 9.0 (3.0%) | 0.0 (0.0%) | 0.248 |
| Neutropenia | None (90+ days) | 178 (59.1%) | 28 (63.6%) | -0.093 | 178.0 (59.1%) | 140.6 (54.9%) | 0.085 |
|  | None (less than 90 days) | 123 (40.9%) | 16 (36.4%) | 0.093 | 123.0 (40.9%) | 115.5 (45.1%) | -0.085 |
|  | Yes | 0 | 0 | - | 0 | 0 | - |
| FN | None (90+ days) | 178 (59.1%) | 28 (63.6%) | -0.093 | 178.0 (59.1%) | 140.6 (54.9%) | 0.085 |
|  | None (less than 90 days) | 123 (40.9%) | 16 (36.4%) | 0.093 | 123.0 (40.9%) | 115.5 (45.1%) | -0.085 |
|  | Yes | 0 | 0 | - | 0 | 0 | - |
| History of surgery for the underlying disease | None (90+ days) | 175 (58.1%) | 27 (61.4%) | -0.066 | 175.0 (58.1%) | 138.1 (53.9%) | 0.085 |
|  | None (less than 90 days) | 122 (40.5%) | 16 (36.4%) | 0.086 | 122.0 (40.5%) | 115.5 (45.1%) | -0.092 |
|  | Yes | 4 (1.3%) | 1 (2.3%) | -0.071 | 4.0 (1.3%) | 2.5 (1.0%) | 0.034 |
| History of surgery for non-primary disease | None (90+ days) | 154 (51.2%) | 24 (54.5%) | -0.068 | 154.0 (51.2%) | 128.5 (50.2%) | 0.020 |
|  | None (less than 90 days) | 103 (34.2%) | 13 (29.5%) | 0.100 | 103.0 (34.2%) | 100.8 (39.4%) | -0.107 |
|  | Yes | 44 (14.6%) | 7 (15.9%) | -0.036 | 44.0 (14.6%) | 26.8 (10.5%) | 0.126 |
| Pre-therapeutic radiotherapy | None (90+ days) | 165 (54.8%) | 24 (54.5%) | 0.005 | 165.0 (54.8%) | 138.7 (54.1%) | 0.013 |
|  | None (less than 90 days) | 108 (35.9%) | 15 (34.1%) | 0.038 | 108.0 (35.9%) | 109.8 (42.9%) | -0.144 |
|  | Yes | 28 (9.3%) | 5 (11.4%) | -0.068 | 28.0 (9.3%) | 7.6 (3.0%) | 0.267 |
| Combined radiotherapy | Nothing | 285 (94.7%) | 38 (86.4%) | 0.287 | 285.0 (94.7%) | 202.2 (79.0%) | 0.478 |
|  | Yes | 16 (5.3%) | 6 (13.6%) | -0.287 | 16.0 (5.3%) | 53.9 (21.0%) | -0.478 |
| Pre-treatment (G-CSF preparation) | None (90+ days) | 178 (59.1%) | 27 (61.4%) | -0.046 | 178.0 (59.1%) | 140.6 (54.9%) | 0.085 |
|  | None (less than 90 days) | 123 (40.9%) | 16 (36.4%) | 0.093 | 123.0 (40.9%) | 115.5 (45.1%) | -0.085 |
|  | Yes | 0 (0.0%) | 1 (2.3%) | -0.216 | 0.0 (0.0%) | 0.0 (0.0%) | 0.000 |
| Pre-treatment (antimicrobial) | None (90+ days) | 174 (57.8%) | 25 (56.8%) | 0.020 | 174.0 (57.8%) | 140.6 (54.9%) | 0.058 |
|  | None (less than 90 days) | 117 (38.9%) | 16 (36.4%) | 0.052 | 117.0 (38.9%) | 115.5 (45.1%) | -0.126 |
|  | Yes | 10 (3.3%) | 3 (6.8%) | -0.160 | 10.0 (3.3%) | 0.0 (0.0%) | 0.262 |
| Previous treatment (anticancer drugs other than lung cancer) | None (90+ days) | 177 (58.8%) | 27 (61.4%) | -0.052 | 177.0 (58.8%) | 140.6 (54.9%) | 0.079 |
|  | None (less than 90 days) | 123 (40.9%) | 16 (36.4%) | 0.093 | 123.0 (40.9%) | 115.5 (45.1%) | -0.085 |
|  | Yes | 1 (0.3%) | 1 (2.3%) | -0.172 | 1.0 (0.3%) | 0.0 (0.0%) | 0.082 |
| Prior treatment (EGFR inhibitor) | None (90+ days) | 118 (39.2%) | 19 (43.2%) | -0.081 | 118.0 (39.2%) | 85.5 (33.4%) | 0.121 |
|  | None (less than 90 days) | 121 (40.2%) | 15 (34.1%) | 0.127 | 121.0 (40.2%) | 113.1 (44.2%) | -0.080 |
|  | Yes | 62 (20.6%) | 10 (22.7%) | -0.052 | 62.0 (20.6%) | 57.5 (22.5%) | -0.045 |
| Prior treatment (ALK inhibitor) | None (90+ days) | 173 (57.5%) | 28 (63.6%) | -0.126 | 173.0 (57.5%) | 140.6 (54.9%) | 0.052 |
|  | None (less than 90 days) | 123 (40.9%) | 16 (36.4%) | 0.093 | 123.0 (40.9%) | 115.5 (45.1%) | -0.085 |
|  | Yes | 5 (1.7%) | 0 (0.0%) | 0.184 | 5.0 (1.7%) | 0.0 (0.0%) | 0.184 |
| Pre-treatment (immune checkpoint inhibitor) | None (90+ days) | 178 (59.1%) | 24 (54.5%) | 0.093 | 178.0 (59.1%) | 140.6 (54.9%) | 0.085 |
|  | None (less than 90 days) | 123 (40.9%) | 16 (36.4%) | 0.093 | 123.0 (40.9%) | 115.5 (45.1%) | -0.085 |
|  | Yes | 0 (0.0%) | 4 (9.1%) | -0.447 | 0.0 (0.0%) | 0.0 (0.0%) | 0.000 |
| Total duration (days) | N. | 301 | 44 | - | 301.0 | 256.1 | - |
|  | Mean +/- SD | 72.8 +/- 41.7 | 89.6 +/- 45.4 | -0.386 | 72.8 +/- 41.7 | 89.0 +/- 120.7 | -0.179 |
|  | Median (Min; Max) | 87.0 (1;181) | 95.5 (10;181) | - | 87.0 (1;181) | 96.0 (10;181) | - |
|  | <30 | 55 (18.3%) | 4 (9.1%) | 0.270 | 55.0 (18.3%) | 24.3 (9.5%) | 0.255 |
|  | 30 ≤, <60 | 66 (21.9%) | 9 (20.5%) | 0.036 | 66.0 (21.9%) | 70.5 (27.5%) | -0.130 |
|  | 60 ≤, <90 | 34 (11.3%) | 4 (9.1%) | 0.073 | 34.0 (11.3%) | 20.6 (8.1%) | 0.110 |
|  | 90 ≤, <120 | 119 (39.5%) | 18 (40.9%) | -0.028 | 119.0 (39.5%) | 79.9 (31.2%) | 0.175 |
|  | 120 ≤ | 27 (9.0%) | 9 (20.5%) | -0.329 | 27.0 (9.0%) | 60.7 (23.7%) | -0.407 |
| Total number of cycles | N. | 301 | 44 | - | 301.0 | 256.1 | - |
|  | Mean +/- SD | 2.8 +/- 1.4 | 3.3 +/- 1.6 | -0.332 | 2.8 +/- 1.4 | 3.3 +/- 4.2 | -0.151 |
|  | Median (Min; Max) | 3.0 (1;6) | 4.0 (1;6) | - | 3.0 (1;6) | 3.0 (1;6) | - |
|  | 1 | 94 (31.2%) | 9 (20.5%) | 0.248 | 94.0 (31.2%) | 56.2 (22.0%) | 0.211 |
|  | 2 | 32 (10.6%) | 5 (11.4%) | -0.023 | 32.0 (10.6%) | 46.9 (18.3%) | -0.219 |
|  | 3 | 35 (11.6%) | 6 (13.6%) | -0.060 | 35.0 (11.6%) | 25.2 (9.8%) | 0.058 |
|  | 4 | 128 (42.5%) | 17 (38.6%) | 0.079 | 128.0 (42.5%) | 67.8 (26.5%) | 0.343 |
|  | 5 | 5 (1.7%) | 2 (4.5%) | -0.167 | 5.0 (1.7%) | 18.1 (7.1%) | -0.266 |
|  | 6 or more | 7 (2.3%) | 5 (11.4%) | -0.364 | 7.0 (2.3%) | 42.0 (16.4%) | -0.498 |
| Atezolizumab total dose | N. | 301 | - | - | 301.0 | - | - |
|  | Mean +/- SD | 3356.8 +/- 1706.4 | - | - | 3356.8 +/- 1706.4 | - | - |
|  | Median (Min; Max) | 3600.0 (1200;7200) | - | - | 3600.0 (1200;7200) | - | - |
| Number of cycles | 1 | 94 (31.2%) | 9 (20.5%) | 0.248 | 94.0 (31.2%) | 56.2 (22.0%) | 0.211 |
|  | 2 | 32 (10.6%) | 5 (11.4%) | -0.023 | 32.0 (10.6%) | 46.9 (18.3%) | -0.219 |
|  | 3 | 35 (11.6%) | 6 (13.6%) | -0.06 | 35.0 (11.6%) | 25.2 (9.8%) | 0.058 |
|  | 4 | 128 (42.5%) | 17 (38.6%) | 0.079 | 128.0 (42.5%) | 67.8 (26.5%) | 0.343 |
|  | 5 | 5 (1.7%) | 2 (4.5%) | -0.167 | 5.0 (1.7%) | 18.1 (7.1%) | -0.266 |
|  | 6 or more | 7 (2.3%) | 5 (11.4%) | -0.364 | 7.0 (2.3%) | 42.0 (16.4%) | -0.498 |
| Bevacizumab total dose | N. | 301 | 44 | - | 301.0 | 256.1 | - |
|  | Mean +/- SD | 2541.2 +/- 1435.2 | 2853.9 +/- 1500.4 | -0.213 | 2541.2 +/- 1435.2 | 2816.2 +/- 3937.3 | -0.093 |
|  | Median (Min; Max) | 2700.0 (400;7200) | 2800.0 (500;6100) | - | 2700.0 (400;7200) | 2800.0 (500;6100) | - |
| Number of cycles | 1 | 94 (31.2%) | 9 (20.5%) | 0.248 | 94.0 (31.2%) | 56.2 (22.0%) | 0.211 |
|  | 2 | 32 (10.6%) | 5 (11.4%) | -0.023 | 32.0 (10.6%) | 46.9 (18.3%) | -0.219 |
|  | 3 | 35 (11.6%) | 6 (13.6%) | -0.06 | 35.0 (11.6%) | 25.2 (9.8%) | 0.058 |
|  | 4 | 128 (42.5%) | 17 (38.6%) | 0.079 | 128.0 (42.5%) | 67.8 (26.5%) | 0.343 |
|  | 5 | 5 (1.7%) | 2 (4.5%) | -0.167 | 5.0 (1.7%) | 18.1 (7.1%) | -0.266 |
|  | 6 or more | 7 (2.3%) | 5 (11.4%) | -0.364 | 7.0 (2.3%) | 42.0 (16.4%) | -0.498 |
| Total carboplatin dose | N. | 301 | 44 | - | 301.0 | 256.1 | - |
|  | Mean +/- SD | 1575.4 +/- 937.4 | 1643.9 +/- 969.7 | -0.072 | 1575.4 +/- 937.4 | 1646.7 +/- 2467.4 | -0.038 |
|  | Median (Min; Max) | 1650.0 (200;3750) | 1500.0 (100;3900) | - | 1650.0 (200;3750) | 1500.0 (100;3900) | - |
| Number of cycles | 1 | 94 (31.2%) | 9 (20.5%) | 0.248 | 94.0 (31.2%) | 56.2 (22.0%) | 0.211 |
|  | 2 | 32 (10.6%) | 5 (11.4%) | -0.023 | 32.0 (10.6%) | 46.9 (18.3%) | -0.219 |
|  | 3 | 35 (11.6%) | 6 (13.6%) | -0.06 | 35.0(11.6%) | 25.2 (9.8%) | 0.058 |
|  | 4 | 128 (42.5%) | 17 (38.6%) | 0.079 | 128.0 (42.5%) | 67.8 (26.5%) | 0.343 |
|  | 5 | 5 (1.7%) | 2 (4.5%) | -0.167 | 5.0 (1.7%) | 18.1 (7.1%) | -0.266 |
|  | 6 or more | 7 (2.3%) | 5 (11.4%) | -0.364 | 7.0 (2.3%) | 42.0 (16.4%) | -0.498 |
| Paclitaxel total dose | N. | 301 | 44 | - | 301.0 | 256.1 | - |
|  | Mean +/- SD | 821.1 +/- 449.9 | 862.0 +/- 473.7 | -0.089 | 821.1 +/- 449.9 | 843.6 +/- 1173.5 | -0.025 |
|  | Median (Min; Max) | 900.0 (200;2400) | 830.0 (60;1980) | - | 900.0 (200;2400) | 800.0 (60;1980) | - |
| Number of cycles | 1 | 94 (31.2%) | 9 (20.5%) | 0.248 | 94.0 (31.2%) | 56.2 (22.0%) | 0.211 |
|  | 2 | 32 (10.6%) | 5 (11.4%) | -0.023 | 32.0 (10.6%) | 46.9 (18.3%) | -0.219 |
|  | 3 | 35 (11.6%) | 6 (13.6%) | -0.06 | 35.0 (11.6%) | 25.2 (9.8%) | 0.058 |
|  | 4 | 128 (42.5%) | 17 (38.6%) | 0.079 | 128.0 (42.5%) | 67.8 (26.5%) | 0.343 |
|  | 5 | 5 (1.7%) | 2 (4.5%) | -0.167 | 5.0 (1.7%) | 18.1 (7.1%) | -0.266 |
|  | 6 or more | 7 (2.3%) | 5 (11.4%) | -0.364 | 7.0 (2.3%) | 42.0 (16.4%) | -0.498 |
| Combination steroids | Nothing | 0 | 0 | - | 0 | 0 | - |
|  | Yes | 301 (100.0%) | 44 (100.0%) | - | 301.0 (100.0%) | 256.1 (100.0%) | - |
| Combined steroid dose (mg) | N. | 301 | 44 | - | 301.0 | 256.1 | - |
|  | Mean +/- SD | 716.9 +/- 864.2 | 662.5 +/- 435.8 | 0.079 | 716.9 +/- 864.2 | 584.5 +/- 953.2 | 0.145 |
|  | Median (Min; Max) | 507.7 (51;8175) | 568.8 (51;1969) | - | 507.7 (51;8175) | 507.7 (51;1969) | - |

Abbreviations: ALK = anaplastic lymphoma kinase; BMI = body mass index; EGFR = epithelial growth factor receptor; FN = febrile neutropenia; G-CSF = granulocyte colony stimulator; Max = maximum; Min = minimum; N = number of cases; SD = standard deviation.

Supplementary Table 6. Sensitivity analysis (cohort design): impact of modifying data enrolment criteria

|  | Exposure group | Control group |
| --- | --- | --- |
| Number of subjects | 301 | 44 |
| Number of FN occurrences | 52 | 2 |
| Incidence rate |  |  |
| Incidence rate per person-year | 0.87 (0.63 - 1.10) | 0.18 (-0.07 - 0.44) |
| Incidence rate ratio (crude) | 4.70 (1.15 - 19.30) | |
| Incidence rate ratio (adjusted) | 7.89 (2.59 - 212,065,719,380.62) | |
| Incidence rate difference (crude) | 0.68 (0.33 - 1.03) | |
| Incidence rate difference (adjusted) | 0.76 (0.46 - 1.01) | |
| Occurrence rate |  |  |
| Occurrence rate (×100%) | 0.17 (0.13 - 0.22) | 0.05 (-0.02 - 0.11) |
| Occurrence rate ratio (crude) | 3.80 (0.96 - 15.05) | |
| Occurrence rate ratio (adjusted) | 6.41 (2.12 - 22.44) | |

Abbreviations: FN = febrile neutropenia.

## **Supplementary Figures**

Supplementary Figure 1. Patient configuration (cohort design).

Patients with NSCLC in MDV

N=387,456

Patients who received the regimens to be investigated as exposure or control group: n=1,650

Patients who started treatment during the inclusion period:

n=1,009

Exposure group

n=301

Control group

n=44

Patients with NSCLC who did not receive regimens of the investigation: n=385,806

Patients who started treatment outside the inclusion period: n=641

Data enrolment record prior to the index date was less than 90 days and no pathological diagnosis existed: n=70

Had chemotherapy for NSCLC before the treatment of investigation: n=383

Regimens did not meet the investigated regimen: n=4

Belonged to the control group in the cohort design: n=207

Abbreviations: MDV = Medical Data Vision; NSCLC = Non-small cell lung cancer.

Supplementary Figure 2. Distribution of trend scores in exposure and control groups for the cohort design A before weighting and B after weighting.

**Supplementary Figure 2A.**


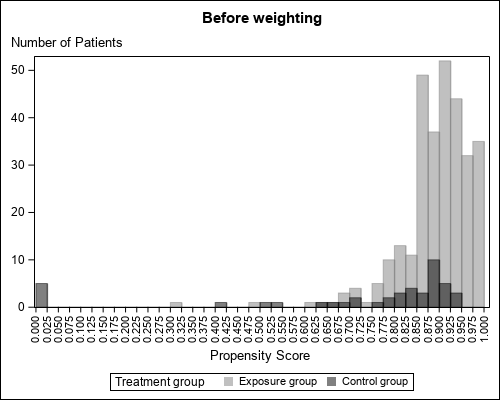


**Supplementary Figure 2B.**


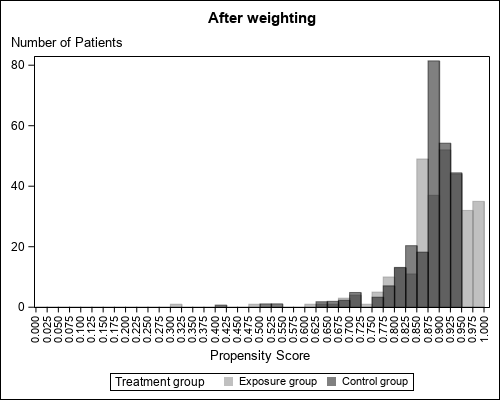

Supplement: Supplementary file 1 — Supplementary file1 (DOCX 304 KB) [file 10147_2024_2669_MOESM1_ESM.docx]
